# Socioeconomic disparities in adolescent anxiety and depression in Finland have not increased during the COVID-19 pandemic

**DOI:** 10.1177/14034948231166466

**Published:** 2023-04-23

**Authors:** Riittakerttu Kaltiala, Terhi Aalto-Setälä, Olli Kiviruusu

**Affiliations:** 1 Tampere University, Faculty of Medicine and Health Technology, Finland; 2 Tampere University Hospital, Department of Adolescent Psychiatry, Finland; 3 Vanha Vaasa Hospital, Finland; 4 Finnish Institute for Health and Welfare, Department of Public Health and Welfare Helsinki, Finland

**Keywords:** COVID-19, depression, anxiety, adolescence, socioeconomic adversity

## Abstract

**Aims:**

The purpose of this research was to assess whether socioeconomic disparities in adolescent depression and anxiety in Finland increased among middle adolescents during the COVID-19 pandemic.

**Methods:**

Repeated cross-sectional surveys (the School Health Promotion Study) from spring 2019 and spring 2021 were compared. The respondents were 87,283 eighth and ninth graders (14–16-year-olds) in 2019 and 91,560 in 2021, corresponding respectively to 73% and 75% of the age groups. Depression was measured by Patient Health Questionnaire-2 (PHQ-2), and anxiety with GAD-7, and adverse socioeconomic background using low parental education, not living with both parents, and family’s poor financial situation. Associations of socioeconomic adversities with depression and generalised anxiety, and the effect of COVID-19 (2021 vs 2019), were analysed using logistic regression.

**Results:**

Depression and anxiety were more common in both sexes the more sociodemographic adversities there were in the adolescent’s background. However, increases in the prevalence of anxiety and depression from pre- to in-pandemic time did not differ with accumulating sociodemographic adversities.

**Conclusions:**

Depression and anxiety increased in prevalence among Finnish adolescents during the pandemic. Sociodemographic disparities in depression and anxiety show no increase. Emotional symptoms are nevertheless more common in adolescents from lower socioeconomic status families.

## Introduction

Mental disorders, particularly depression and anxiety, have increased worldwide during the COVID-19 pandemic among both adults [1,2] and adolescents [3]. Pandemic-related factors likely to have a negative influence on mental health and psychological well-being include economic hardship, social isolation and distancing, and concern about significant others’ and one’s own health. For adolescents, social isolation due to quarantines, lockdowns and social distancing is likely to create a particular source of stress, given the considerable significance of interactions with peers during this developmental phase [4]. Remote schooling, with increased pressures to accomplish academic work independently, the collapse of daily routines and schedules, missed milestones, reduced exercise, excessive social media use and exposure to increased parental stress are potential reasons for declining mental health among adolescents during the pandemic [3,5]. The later in the course of the pandemic emotional symptoms in adolescent populations were assessed, the more prevalent they were [3].

Lower socioeconomic status (SES), measured by income, education, occupational status or unemployment, has been associated with emotional disorders among adults and adolescents alike, and the negative impact on people who experienced disadvantaged socioeconomic status in early life persists into adulthood [6–8]. These aspects of socioeconomic disadvantage may relate to emotional disorders through economic stress, lack of social capital and stressors related to a disadvantaged living environment. The disadvantaged socioeconomic status of the family may influence young people in multiple pathways. These include material hardship, relational poverty and social exclusion, the impact of disadvantaged living conditions and school, parental stress impairing parent–adolescent relationships and fewer parental resources to support adolescent development [9].

Among adults, the findings concerning trends in depression and anxiety according to socioeconomic factors during the COVID-19 pandemic have been inconsistent. Some research has suggested that as people of lower socioeconomic statuses have suffered the greatest risk of job loss and economic hardship, worries created by jobs not allowing the safety of remote working and the stress created by cramped living conditions, the increases in depression and anxiety over the pandemic period have been greater among those under greater financial stress and/or with low SES [10–12]. Others have found familiar SES disparities but no changes in relative status between SES groups [1,2], while others have reported that exacerbation of emotional symptoms has been greatest among those with the highest socioeconomic status [13–15]. Moreover, increases in depression and anxiety have been reportedly greater among younger adults and women [1,2,14,15]. Among adolescents, Waite et al. [16] reported no increase in socioeconomic disparities in adolescent depression from before to during the pandemic but conceded that the sample was biased towards more affluent families. Hafstad et al. [17] observed that increases in anxiety and depression during the pandemic were greater among adolescents living in single-parent families but smaller among adolescents perceiving their family’s situation as poor before the pandemic, thereby leaving the role of SES in this matter inconclusive.

In sum, emotional disorders and symptoms, particularly depression and anxiety, have reportedly increased in both adults and adolescents during the COVID-19 pandemic. This has also been observed in Finland [18], with mental health problems more common among lower SES groups across ages. The effects of hardships related to the COVID-19 pandemic have been more severe among families with low SES than among those with higher SES. This suggests that disparities in mental well-being contingent upon SES may have increased during the pandemic. However, the findings concerning changes in socioeconomic disparities in mental health among adults have been contradictory. Increasing SES disparities in emotional disorders during the pandemic have been suggested among children and adolescents [5], but to the best of our knowledge few studies have actually analysed whether increases in depression and anxiety among adolescents have indeed differed by SES.

## Aims

The aim of the present study was to explore whether the known socioeconomic disparities in adolescent depression and anxiety increased during the COVID-19 pandemic among middle adolescents in Finland. The data were analysed stratified by sex because, starting from puberty, depression and anxiety are more common in females than males and increases in these problems during the pandemic have been found to have been greater in adolescent females [19,20].

## Methods

The School Health Promotion Study (SHPS) is a nationwide, school-based cross-sectional anonymous survey designed to examine the health, health behaviours and school experiences of teenagers across Finland. It is conducted biannually by the Finnish Institute for Health and Welfare (THL). The survey is run primarily for health policy and administrative purposes, with the main aim of producing adolescent health indicators at national, municipal and school levels. The questions posed are formulated by a panel of experts in the THL. Researchers can apply to the national data authorities for permission to use the data for purposes of scientific research. The authors of the present study did not design the study but obtained the data through the appropriate applications.

The survey is sent to every municipality in Finland and the municipalities decide if the schools in their areas will participate. Parents are informed about the survey but, according to the child ombudsman’s ruling of 2012, children are at liberty to express their opinions in the SHPS at their own discretion. The pupils are duly informed about the voluntary nature of participation and about their option to leave any questions unanswered or to cease participating at any point. They then respond to the questionnaire online during a school lesson supervised by a teacher, who does not interfere with responding but ensures that they all have peace to respond undisturbed. Completion of the questionnaire is taken to be consent to participate. The survey is implemented primarily for health policy and administrative purposes and the data is available on request for purposes of scientific research. The School Health Promotion Study has received ethical approval from the institutional review board of the Finnish Institute of Health and Welfare.

The survey is conducted among fourth, fifth, eighth and ninth graders of comprehensive schools, and among second-year students in upper secondary education, during the spring term. The present analyses used SHPS data on eighth and ninth graders from the years 2019 (pre-pandemic) and 2021 (during the pandemic). Nine-year comprehensive education, starting at age seven, is compulsory in Finland. The coverage rate among eight and ninth graders was 73% (*N*=87,283) in 2019 and 75% (*N*=91,560) in 2021 [21]. The respondents are described in Table I.

**Table I.** Study participants and descriptive statistics by sex and study year.

	Boys, *N*=86,547			Girls, *N*=90,368		
	2019 *N*=42,468	2021 *N*=44,079		2019 *N*=43,899	2021 *N*=46,469	
	%	%	*p* ^a^	%	%	*p* ^a^
Age, mean (SD)	15.3 (0.7)	15.3 (0.6)	0.002	15.3 (0.6)	15.3 (0.6)	0.267
Depression (PHQ-2 ⩾ 3)			<0.001			<0.001
No	89.4	86.4		77.8	68.2	
Yes	8.1	10.3		20.8	30.0	
Missing	2.5	3.2		1.4	1.8	
Generalised anxiety (GAD-7 ⩾ 10)			<0.001			<0.001
No	93.7	91.0		80.1	69.6	
Yes	4.9	7.2		19.5	29.8	
Missing	1.4	1.8		0.4	0.6	
Lives with both parents			0.041			<0.001
Yes	65.4	64.9		67.0	65.5	
No	26.4	27.0		29.9	31.0	
Missing	8.2	8.1		3.2	3.5	
Both parents only basic education			0.333			0.566
No	87.3	88.5		92.0	92.7	
Yes	2.1	2.3		2.2	2.3	
Missing	10.6	9.3		5.8	5.0	
Family’s financial situation			0.427			<0.001
Good	72.8	73.5		69.6	69.0	
Moderate	16.5	16.8		22.3	23.4	
Poor	3.5	3.4		5.6	5.2	
Missing	7.3	6.4		2.5	2.4	
Sum-index of socioeconomic adversities			0.228			<0.001
0	50.3	50.7		48.6	47.6	
1	25.9	26.8		29.0	30.4	
2	7.9	8.2		11.6	12.1	
3	1.7	1.7		3.0	2.9	
4	0.1	0.2		0.1	0.1	
Missing	14.1	12.4		7.7	7.0	

a Difference between years tested with ANOVA for age, otherwise with chi-square test; tests based only on valid values, missing values excluded.

### Measures

#### Depression

Depression was measured with Patient Health Questionnaire-2 (PHQ-2), a self-report screening tool with two questions: ‘During the past two weeks, how often have you been bothered by (1) feeling down, depressed, or hopeless? (2) little interest or pleasure in doing things?’ Response options for both questions were ‘not at all’, ‘for several days’, ‘for more than half the days’ and ‘nearly every day’. The PHQ-2 has been shown to be a reliable method for detecting depression in adolescents and young adults [22,23]. In the analyses, the sum score of the two items was dichotomised to depression (3 or more points) vs no depression (<3).

#### Anxiety

Generalized anxiety symptoms were elicited with the GAD-7, a self-report questionnaire designed to identify probable cases of generalised anxiety disorder and to assess symptom severity. The GAD-7 items describe the most prominent diagnostic features of the DSM-IV generalised anxiety disorder. The GAD-7 elicits how often during the last two weeks the respondent has been bothered by each of the seven core symptoms of generalised anxiety disorder. Response options are ‘not at all’, ‘for several days’, ‘for more than half the days’ and ‘nearly every day’, scored respectively as 0, 1, 2 and 3. The GAD-7 has been shown to be a reliable and valid measure for detecting generalised anxiety disorder in primary care and general population [24]. In the analyses, the sum score of these seven items was dichotomised to moderate or severe anxiety (10 + points) vs no anxiety (<10). Correlation between depression and anxiety was *r*=0.61.

#### Sex

Respondents reported their official sex (boy or girl).

#### Living with both parents

Respondents reported whether they were living in a household with both parents (yes = 0/no = 1).

#### Both parents with only basic education

Parental education (separately for mother and father) was elicited with the question ‘What is the highest educational level your parents have achieved?’ with four response options: ‘comprehensive school or equivalent’, ‘upper secondary school or vocational school’, ‘vocational studies in addition to upper secondary school’, and ‘university, university of applied sciences or other higher education institution’. For the analyses, a dichotomised variable was constructed indicating that (1) both parents had only basic education (comprehensive school or equivalent) vs other (0).

#### Family’s financial situation

The family’s financial situation was elicited with the question ‘How would you rate your family’s financial situation?’ and the response options were: ‘very good’, ‘fairly good’, ‘moderate’, ‘fairly poor’ and ‘very poor’. For the analyses, these were recoded into a three-category variable: 0 = ‘good’ (very/fairly good); 1 = ‘moderate’ (moderate); 2 = ‘poor’ (fairly/very poor).

#### Sum-index of socioeconomic adversities

The sum-index of socioeconomic adversities was calculated as the sum of the three socioeconomic adversities, with a score of 0 indicating no adversities (i.e. living with both parents, at least one parent with higher than basic education, good family financial situation) and a score of 4 indicating that the respondent does not live with both parents, both parents have only basic education and that the family’s financial situation is poor.

### Statistical analyses

All analyses were done separately for boys and girls using SPSS 28.0 software. Missing values on sex resulted in the exclusion of 487 cases (0.3%). Further, 1,373 cases (0.8%) were excluded from the analyses due to suspected unreliable (or facetious) responding. This was assessed using three questions on disabilities, and respondents reporting they were ‘completely unable’ to see, hear and walk were considered unreliable. If their responses to the disabilities question had been truthful, they would be highly unlikely to be attending schools participating in the SHPS or indeed able to respond to the questionnaire at all (see also [25]). After these exclusions, there were 176,915 cases left for the analyses. Percentages of missing data for the study variables are given in Table I.

Distributions of the study variables by sex and year of study are presented first. Differences between years were tested with analysis of variance (ANOVA) for continuous and with chi-square test for categorical variables. Next, we examined whether the socioeconomic adversities of interest were associated with depression and generalised anxiety. These analyses, conducted using logistic regression, were done separately for each socioeconomic adversity variable and using the total sample, i.e. years 2019 and 2021 combined. For odds ratios (OR), 95% confidence intervals (CI) were calculated. Finally, to determine whether the effects of the COVID-19 pandemic on the prevalence of depression and generalized anxiety were different in the categories of socioeconomic adversity variables, separate logistic regression models were run for each such category and the effect of the COVID-19 pandemic time (coded as ‘1’ for 2021, ‘0’ for 2019) was reported for each. Differences in the effects between categories (i.e. moderation) were tested using COVID-19 × socioeconomic adversity variable interaction terms. Significant interaction would indicate that changes from 2019 to 2021 (here labelled as the effect of COVID-19) differed between the categories of socioeconomic adversity. Interaction effects were analysed separately for each socioeconomic adversity variable, the model containing only effects for COVID-19, socioeconomic adversity and their interaction (see supplemental material). In the logistic regression models, the last two categories of the sum-index of socioeconomic adversities were grouped together due to small cell frequencies and increased confidence intervals.

## Results

The mean age of the participants was 15.3 years (Table I). There were only minor changes in the socioeconomic adversity indicators from 2019 to 2021, although some of these were statistically significant. Among boys there was more missing information on socioeconomic adversity measures than among girls.

Not living with both parents, both parents having only basic education and poorer financial situation of the family were all associated with increased odds of having depression and generalised anxiety (Table II). Having multiple adversities was also associated with higher probability of emotional symptoms. All associations were significant for boys and girls.

**Table II.** Bivariate associations of socioeconomic adversities with depression and generalised anxiety.^a^

Variable	Category	Boys		Girls	
		OR	95% CI	OR	95% CI
***Dependent: depression***					
Lives with both parents	Yes (ref.)	1.00		1.00	
	No	1.59	1.52–1.67	1.56	1.51–1.61
Both parents only basic education	No (ref.)	1.00		1.00	
	Yes	1.88	1.65–2.13	1.28	1.16–1.41
Family’s financial situation	Good (ref.)	1.00		1.00	
	Moderate	2.28	2.15–2.41	2.17	2.09–2.24
	Poor	5.15	4.72–5.61	3.85	3.63–4.09
Sum-index of socioeconomic adversities	0 (ref.)	1.00		1.00	
	1	1.70	1.60–1.80	1.62	1.56–1.68
	2	3.08	2.86–3.32	2.84	2.72–2.98
	3–4	6.21	5.54–6.96	4.41	4.08–4.77
***Dependent: generalised anxiety***					
Lives with both parents	Yes (ref.)	1.00		1.00	
	No	1.60	1.51–1.70	1.47	1.42–1.51
					
Both parents only basic education	No (ref.)	1.00		1.00	
	Yes	2.32	2.01–2.67	1.21	1.10–1.34
Family’s financial situation	Good (ref.)	1.00		1.00	
	Moderate	2.16	2.02–2.31	2.01	1.94–2.08
	Poor	5.19	4.71–5.73	3.57	3.36–3.79
Sum-index of socioeconomic adversities	0 (ref.)	1.00		1.00	
	1	1.61	1.50–1.73	1.53	1.47–1.58
	2	3.08	2.82–3.36	2.53	2.42–2.65
	3–4	6.38	5.61–7.25	3.96	3.66–4.28

a Separate models for each bivariate association between a socioeconomic adversity and mental health variable. Total sample, years 2019 and 2021 combined analysed (*N*=176,915).

Among boys, the OR of COVID-19 (2021) for depression was 1.33 (95% CI: 1.27–1.39) and for generalised anxiety 1.52 (1.43–1.61) (Table III). There was only a little variation in these ORs when analysed separately in the different categories of socioeconomic adversities, and there were no statistically significant interactions to suggest differences in the effects between categories. For example, regarding family’s financial situation, in spite of some differences in the ORs of COVID-19 (change from 2019 to 2021) for depression (1.42, 1.30 and 1.28 for the categories of good, moderate and poor), the interaction term COVID-19 × financial situation was not statistically significant (Supplemental Table S9), indicating that the ORs were not different, and hence no differences in the effects of COVID-19 on depression by family’s financial situation were suggested. Among girls, the ORs of COVID-19 were 1.64 (95% CI: 1.59–1.69) and 1.76 (1.70–1.81) for depression and generalised anxiety respectively. Again, the variation in the ORs between categories was relatively modest, while there were some statistically significant interactions. Those girls in families with a good financial situation and with no socioeconomic adversities showed a stronger effect of COVID-19 on generalised anxiety (Table III, Supplemental Tables S12 and S16).

**Table III.** The effect of COVID-19 (2021 vs 2019) on depression and generalised anxiety in subgroups of socioeconomic adversity.^a^

	Group	Boys		Girls	
		OR	95% CI	OR	95% CI
***Dependent: depression***					
Total	All	1.33	1.27–1.39	1.64	1.59–1.69
Lives with both parents	Yes	1.36	1.28–1.45	1.66	1.60–1.72
	No	1.34	1.24–1.45	1.63	1.55–1.72
Both parents only basic education	No	1.36	1.29–1.43	1.66	1.60–1.71
	Yes	1.31	1.02–1.68	1.63	1.34–1.98
Family’s financial situation	Good	1.42	1.33–1.51	1.73	1.66–1.80
	Moderate	1.30	1.19–1.43	1.61	1.52–1.71
	Poor	1.28	1.09–1.50	1.64	1.46–1.84
Sum-index of socioeconomic adversities	0	1.45	1.34–1.56	1.70	1.62–1.79
	1	1.31	1.21–1.43	1.67	1.58–1.76
	2	1.43	1.26–1.62	1.60	1.48–1.73
	3–4	1.20	0.97–1.49	1.69	1.45–1.97
***Dependent: generalised anxiety***					
Total	All	1.52	1.43–1.61	1.76	1.70–1.81
Lives with both parents	Yes	1.54	1.42–1.66	1.80	1.73–1.87
	No	1.56	1.41–1.72	1.71	1.62–1.80
Both parents only basic education	No	1.56	1.46–1.66	1.77	1.72–1.83
	Yes	1.45	1.10–1.92	1.66	1.36–2.02
Family’s financial situation	Good	1.63	1.50–1.76	1.88^c^	1.80–1.95
	Moderate	1.51	1.34–1.69	1.69^c^	1.59–1.79
	Poor	1.48	1.23–1.78	1.70	1.51–1.90
Sum-index of socioeconomic adversities	0	1.60	1.45–1.77	1.90^b,c^	1.81–2.00
	1	1.57	1.40–1.75	1.73^b^	1.63–1.83
	2	1.52	1.31–1.77	1.66^c^	1.53–1.80
	3–4	1.59	1.25–2.03	1.80	1.55–2.10

a A separate model for each category of each socioeconomic adversity.

Statistical significance of the interaction term comparing the effects with same superscription: ^b^*p* < 0.05; ^c^*p* < 0.01. Interactions are from models where socioeconomic adversity, COVID-19 and socioeconomic adversity × COVID-19 interaction predict the dependent mental health variable; a separate model was conducted for each socioeconomic adversity variable (see supplemental material).

## Discussion

In this large repeated-surveys study representative of Finnish adolescents aged 14–16, prevalence of depression and anxiety increased among both boys and girls from spring 2019 (before COVID-19) to spring 2021 (during COVID-19). In both sexes, depression and anxiety were associated with all the indicators of family’s adverse socioeconomic status studied and were more common the more adversities accumulated. However, contrary to expectations, increases in prevalence of depression and anxiety were not greater among adolescents from less privileged socioeconomic backgrounds. Nor did socioeconomic disparities in adolescent depression and anxiety increase in the course of the pandemic. Actually, regarding anxiety among girls, a slightly greater increase was seen in the socioeconomically most privileged group.

Given that many pandemic-associated factors likely have a greater negative impact on families with lower socioeconomic status, increasing socioeconomic disparities in depression and anxiety during the time of the pandemic were to be expected. However, these were not found. Among adults, greater increases in emotional symptoms have even been reported among higher SES groups [13–15], although not systematically [1,2,10–12]. Our only finding on changes in the relative positions of SES groups was the slightly greater increase in anxiety among girls from the most privileged SES group. Depression and anxiety were nevertheless strongly associated with accumulating socioeconomic adversities, and this disparity persisted almost consistently. Earlier reports concerning the role of SES in changes in adolescent mental health from before to during the pandemic have been inconsistent [16,17]. In conclusion, it is currently not justified to claim that negative changes in adolescents’ mental health during the COVID-19 pandemic were greater in adolescents with lower SES background. However, as symptom levels were nevertheless more prevalent among youth from disadvantaged SES background, health and social policies need to focus on reducing the disparities in the mental health of young people attributable to SES.

Similarly, a counterintuitive observation was reported by Hawke et al. [26] that youth in a community sample suffered greater pandemic-related increases in emotional and behavioural symptoms than youth in a clinical sample, even if the latter are likely the most vulnerable youth with least resilience when facing hardship. In another study including both clinical and community sample youth [27], the parents of the clinically treated children and adolescents reported no increases in their children’s internalising symptoms during the pandemic, even if such changes were reported by the young people themselves. Such counterintuitive findings could relate to a ceiling effect, in that the clinically treated young people already had high levels of symptoms to start with, and thus there was possibly less room for deterioration, or less sensitivity remaining in symptom measures to detect further deterioration [26,27]. In our data, symptom levels among the most disadvantaged youth were already six-fold among boys and four-fold among girls in the pre-pandemic situation compared to those living with both parents, and children of parents with the highest levels of education perceived their family’s economic situation to be good.

### Methodological considerations

Using two large nationwide samples of 14–16-year-old adolescents is a strength of the present study. The coverage of compulsory comprehensive education in Finland is almost 99%. The study did not reach adolescents who were not at school on the survey day. Such absence is known to amount to 10–15% of those enrolled in schools. Among those absent, psychosocial problems of all kinds may be more common than among attending pupils [28]. However, not even major attrition need necessarily bias the conclusions on the relationships between the phenomena studied in survey studies [29].

Depression and anxiety were measured with validated instruments suitable for use among adolescent populations [22,24]. The same instruments have been used worldwide in many studies assessing depression and anxiety among adolescents during the pandemic [3]. Predictably, the symptom levels and changes therein were also comparable to internationally reported figures [3].

A limitation is that the design of repeated cross-sectional surveys does not permit analysis of within-individual changes. The clear increase in symptoms in the course of the pandemic, also seen globally [3], suggests the impact of the pandemic, but other reasons for changes from 2019 to 2021 cannot be ruled out. Questions directly related to the pandemic were not included, which is a limitation.

## Conclusion

Adolescent depression and anxiety increased in Finland during the COVID-19 pandemic, but even if many consequences of the pandemic and the measures for controlling it may have a more severe impact on lower SES families, socioeconomic disparities in adolescent emotional symptoms did not increase. The decline in adolescent mental health thus likely relates more to the effect of the pandemic control measures on the adolescents’ personal lives than to SES adversities within the family. Nevertheless, adolescents from low SES families suffer disproportionately from depression and anxiety. Health and social policies need to focus on reducing these disparities even if they have not further increased during the pandemic.

## Supplemental Material

sj-docx-1-sjp-10.1177_14034948231166466 – Supplemental material for Socioeconomic disparities in adolescent anxiety and depression in Finland have not increased during the COVID-19 pandemic Click here for additional data file. Supplemental material, sj-docx-1-sjp-10.1177_14034948231166466 for Socioeconomic disparities in adolescent anxiety and depression in Finland have not increased during the COVID-19 pandemic by Riittakerttu Kaltiala, Terhi Aalt-Setälä and Olli Kiviruusu in Scandinavian Journal of Public Health
