# Supplementary material for: Socioeconomic disparities in adolescent anxiety and depression in Finland have not increased during the COVID-19 pandemic
Source: Scand J Public Health. 2023 Apr 23;51(5):656–63. doi: 10.1177/14034948231166466 (PMC10125881; doi:10.1177/14034948231166466)
Supplement: sj-docx-1-sjp-10.1177_14034948231166466 – Supplemental material for Socioeconomic disparities in adolescent anxiety and depression in Finland have not increased during the COVID-19 pandemic [file sj-docx-1-sjp-10.1177_14034948231166466.docx]

**Supplementary material**

**Logistic regression models of interactions between COVID-19 (year 2021 vs 2019) and socioeconomic adversities on adolescent depression and generalized anxiety by sex.**

Table S1. Logistic regression model with COVID-19 (year 2021; year 2019 as the reference category), living with both parents (“No”; “Yes” as the reference category), and their interaction predicting depression (PHQ-2 ≥ 3) in boys.

| **Variables in the Equation^a^** | | | | | | | | | |
| --- | --- | --- | --- | --- | --- | --- | --- | --- | --- |
|  | | B | S.E. | Wald | df | Sig. | Exp(B) | 95% C.I.for EXP(B) | |
|  |  |  |  |  |  |  |  | Lower | Upper |
| Step 1^b^ | Year 2021 | .306 | .032 | 94.231 | 1 | .000 | 1.358 | 1.277 | 1.445 |
|  | Living with both parents (No) | .472 | .039 | 146.489 | 1 | .000 | 1.603 | 1.485 | 1.731 |
|  | Year 2021 by Living with both parents (No) | -.014 | .052 | .073 | 1 | .787 | .986 | .891 | 1.091 |
|  | Constant | -2.598 | .024 | 11889.645 | 1 | .000 | .074 |  |  |
| a. Sex = Boy | | | | | | | | | |
| b. Variable(s) entered on step 1: year 2021, living with both parents, year 2021 * living with both parents. | | | | | | | | | |

Table S2. Logistic regression model with COVID-19 (year 2021; year 2019 as the reference category), living with both parents (“No”; “Yes” as the reference category), and their interaction predicting depression (PHQ-2 ≥ 3) in girls.

| **Variables in the Equation^a^** | | | | | | | | | |
| --- | --- | --- | --- | --- | --- | --- | --- | --- | --- |
|  | | B | S.E. | Wald | df | Sig. | Exp(B) | 95% C.I.for EXP(B) | |
|  |  |  |  |  |  |  |  | Lower | Upper |
| Step 1^b^ | Year 2021 | .506 | .020 | 650.157 | 1 | .000 | 1.658 | 1.595 | 1.724 |
|  | Living with both parents (No) | .451 | .025 | 326.822 | 1 | .000 | 1.570 | 1.495 | 1.649 |
|  | Year 2021 by Living with both parents (No) | -.015 | .033 | .214 | 1 | .644 | .985 | .923 | 1.051 |
|  | Constant | -1.469 | .015 | 9536.721 | 1 | .000 | .230 |  |  |
| a. Sex = Girl | | | | | | | | | |
| b. Variable(s) entered on step 1: year 2021, living with both parents, year 2021 * living with both parents. | | | | | | | | | |

Table S3. Logistic regression model with COVID-19 (year 2021; year 2019 as the reference category), living with both parents (“No”; “Yes” as the reference category), and their interaction predicting generalized anxiety (GAD-7 ≥ 10) in boys.

| **Variables in the Equation^a^** | | | | | | | | | |
| --- | --- | --- | --- | --- | --- | --- | --- | --- | --- |
|  | | B | S.E. | Wald | df | Sig. | Exp(B) | 95% C.I.for EXP(B) | |
|  |  |  |  |  |  |  |  | Lower | Upper |
| Step 1^b^ | Year 2021 | .429 | .039 | 119.936 | 1 | .000 | 1.536 | 1.423 | 1.659 |
|  | Living with both parents (No) | .461 | .049 | 86.790 | 1 | .000 | 1.585 | 1.439 | 1.747 |
|  | Year 2021 by Living with both parents (No) | .015 | .064 | .055 | 1 | .815 | 1.015 | .896 | 1.150 |
|  | Constant | -3.158 | .030 | 10779.572 | 1 | .000 | .043 |  |  |
| a. Sex = Boy | | | | | | | | | |
| b. Variable(s) entered on step 1: year 2021, living with both parents, year 2021 * living with both parents. | | | | | | | | | |

Table S4. Logistic regression model with COVID-19 (year 2021; year 2019 as the reference category), living with both parents (“No”; “Yes” as the reference category), and their interaction predicting generalized anxiety (GAD-7 ≥ 10) in girls.

| **Variables in the Equation^a^** | | | | | | | | | |
| --- | --- | --- | --- | --- | --- | --- | --- | --- | --- |
|  | | B | S.E. | Wald | df | Sig. | Exp(B) | 95% C.I.for EXP(B) | |
|  |  |  |  |  |  |  |  | Lower | Upper |
| Step 1^b^ | Year 2021 | .588 | .020 | 861.199 | 1 | .000 | 1.801 | 1.731 | 1.873 |
|  | Living with both parents (No) | .410 | .026 | 256.358 | 1 | .000 | 1.506 | 1.432 | 1.584 |
|  | Year 2021 by Living with both parents (No) | -.052 | .034 | 2.366 | 1 | .124 | .950 | .889 | 1.014 |
|  | Constant | -1.554 | .015 | 10204.571 | 1 | .000 | .211 |  |  |
| a. Sex = Girl | | | | | | | | | |
| b. Variable(s) entered on step 1: year 2021, living with both parents, year 2021 * living with both parents. | | | | | | | | | |

Table S5. Logistic regression model with COVID-19 (year 2021; year 2019 as the reference category), both parents only basic education (“Yes”; “No” as the reference category), and their interaction predicting depression (PHQ-2 ≥ 3) in boys.

| **Variables in the Equation^a^** | | | | | | | | | |
| --- | --- | --- | --- | --- | --- | --- | --- | --- | --- |
|  | | B | S.E. | Wald | df | Sig. | Exp(B) | 95% C.I.for EXP(B) | |
|  |  |  |  |  |  |  |  | Lower | Upper |
| Step 1^b^ | Year 2021 | .308 | .026 | 141.994 | 1 | .000 | 1.361 | 1.293 | 1.431 |
|  | Both parents only basic education (Yes) | .651 | .099 | 43.555 | 1 | .000 | 1.918 | 1.581 | 2.328 |
|  | Year 2021 by Both parents only basic education (Yes) | -.039 | .131 | .088 | 1 | .766 | .962 | .744 | 1.244 |
|  | Constant | -2.479 | .020 | 16022.410 | 1 | .000 | .084 |  |  |
| a. Sex = Boy | | | | | | | | | |
| b. Variable(s) entered on step 1: year 2021, both parents only basic education, year 2021 * both parents only basic education. | | | | | | | | | |

Table S6. Logistic regression model with COVID-19 (year 2021; year 2019 as the reference category), both parents only basic education (“Yes”; “No” as the reference category), and their interaction predicting depression (PHQ-2 ≥ 3) in girls.

| **Variables in the Equation^a^** | | | | | | | | | |
| --- | --- | --- | --- | --- | --- | --- | --- | --- | --- |
|  | | B | S.E. | Wald | df | Sig. | Exp(B) | 95% C.I.for EXP(B) | |
|  |  |  |  |  |  |  |  | Lower | Upper |
| Step 1^b^ | Year 2021 | .504 | .016 | 963.990 | 1 | .000 | 1.655 | 1.603 | 1.709 |
|  | Both parents only basic education (Yes) | .256 | .076 | 11.366 | 1 | .001 | 1.292 | 1.113 | 1.500 |
|  | Year 2021 by Both parents only basic education (Yes) | -.016 | .100 | .026 | 1 | .873 | .984 | .808 | 1.198 |
|  | Constant | -1.338 | .012 | 11782.215 | 1 | .000 | .262 |  |  |
| a. Sex = Girl | | | | | | | | | |
| b. Variable(s) entered on step 1: year 2021, both parents only basic education, year 2021 * both parents only basic education. | | | | | | | | | |

Table S7. Logistic regression model with COVID-19 (year 2021; year 2019 as the reference category), both parents only basic education (“Yes”; “No” as the reference category), and their interaction predicting generalized anxiety (GAD-7 ≥ 10) in boys.

| **Variables in the Equation^a^** | | | | | | | | | |
| --- | --- | --- | --- | --- | --- | --- | --- | --- | --- |
|  | | B | S.E. | Wald | df | Sig. | Exp(B) | 95% C.I.for EXP(B) | |
|  |  |  |  |  |  |  |  | Lower | Upper |
| Step 1^b^ | Year 2021 | .442 | .032 | 189.317 | 1 | .000 | 1.556 | 1.461 | 1.657 |
|  | Both parents only basic education (Yes) | .880 | .113 | 60.820 | 1 | .000 | 2.411 | 1.933 | 3.008 |
|  | Year 2021 by Both parents only basic education (Yes) | -.070 | .147 | .225 | 1 | .635 | .933 | .700 | 1.243 |
|  | Constant | -3.053 | .025 | 14818.335 | 1 | .000 | .047 |  |  |
| a. Sex = Boy | | | | | | | | | |
| b. Variable(s) entered on step 1: year 2021, both parents only basic education, year 2021 * both parents only basic education. | | | | | | | | | |

Table S8. Logistic regression model with COVID-19 (year 2021; year 2019 as the reference category), both parents only basic education (“Yes”; “No” as the reference category), and their interaction predicting generalized anxiety (GAD-7 ≥ 10) in girls.

| **Variables in the Equation^a^** | | | | | | | | | |
| --- | --- | --- | --- | --- | --- | --- | --- | --- | --- |
|  | | B | S.E. | Wald | df | Sig. | Exp(B) | 95% C.I.for EXP(B) | |
|  |  |  |  |  |  |  |  | Lower | Upper |
| Step 1^b^ | Year 2021 | .573 | .016 | 1214.769 | 1 | .000 | 1.773 | 1.717 | 1.831 |
|  | Both parents only basic education (Yes) | .231 | .078 | 8.862 | 1 | .003 | 1.260 | 1.082 | 1.467 |
|  | Year 2021 by Both parents only basic education (Yes) | -.068 | .102 | .450 | 1 | .502 | .934 | .764 | 1.141 |
|  | Constant | -1.427 | .013 | 12820.413 | 1 | .000 | .240 |  |  |
| a. Sex = Girl | | | | | | | | | |
| b. Variable(s) entered on step 1: year 2021, both parents only basic education, year 2021 * both parents only basic education. | | | | | | | | | |

Table S9. Logistic regression model with COVID-19 (year 2021; year 2019 as the reference category), family’s financial situation (”Good” as the reference category), and their interaction predicting depression (PHQ-2 ≥ 3) in boys.

| **Variables in the Equation^a^** | | | | | | | | | |
| --- | --- | --- | --- | --- | --- | --- | --- | --- | --- |
|  | | B | S.E. | Wald | df | Sig. | Exp(B) | 95% C.I.for EXP(B) | |
|  |  |  |  |  |  |  |  | Lower | Upper |
| Step 1^b^ | Year 2021 | .349 | .032 | 121.387 | 1 | .000 | 1.418 | 1.333 | 1.509 |
|  | Family's financial situation |  |  | 923.236 | 2 | .000 |  |  |  |
|  | Family's financial situation (Moderate) | .872 | .043 | 407.173 | 1 | .000 | 2.392 | 2.197 | 2.603 |
|  | Family's financial situation (Poor) | 1.706 | .064 | 700.528 | 1 | .000 | 5.505 | 4.852 | 6.246 |
|  | Year 2021 * Family's financial situation |  |  | 3.027 | 2 | .220 |  |  |  |
|  | Year 2021 by Family's financial situation (Moderate) | -.084 | .057 | 2.166 | 1 | .141 | .919 | .821 | 1.028 |
|  | Year 2021 by Family's financial situation (Poor) | -.106 | .088 | 1.445 | 1 | .229 | .899 | .756 | 1.069 |
|  | Constant | -2.763 | .024 | 12974.668 | 1 | .000 | .063 |  |  |
| a. Sex = Boy | | | | | | | | | |
| b. Variable(s) entered on step 1: year 2021, family’s financial situation, year 2021 * family’s financial situation. | | | | | | | | | |

Table S10. Logistic regression model with COVID-19 (year 2021; year 2019 as the reference category), family’s financial situation (”Good” as the reference category), and their interaction predicting depression (PHQ-2 ≥ 3) in girls.

| **Variables in the Equation^a^** | | | | | | | | | |
| --- | --- | --- | --- | --- | --- | --- | --- | --- | --- |
|  | | B | S.E. | Wald | df | Sig. | Exp(B) | 95% C.I.for EXP(B) | |
|  |  |  |  |  |  |  |  | Lower | Upper |
| Step 1^b^ | Year 2021 | .546 | .020 | 722.872 | 1 | .000 | 1.727 | 1.660 | 1.797 |
|  | Family's financial situation |  |  | 1611.418 | 2 | .000 |  |  |  |
|  | Family's financial situation (Moderate) | .816 | .027 | 909.905 | 1 | .000 | 2.262 | 2.146 | 2.386 |
|  | Family's financial situation (Poor) | 1.405 | .044 | 1032.987 | 1 | .000 | 4.076 | 3.741 | 4.440 |
|  | Year 2021 * Family's financial situation |  |  | 4.045 | 2 | .132 |  |  |  |
|  | Year 2021 by Family's financial situation (Moderate) | -.070 | .036 | 3.822 | 1 | .051 | .932 | .869 | 1.000 |
|  | Year 2021 by Family's financial situation (Poor) | -.051 | .062 | .688 | 1 | .407 | .950 | .842 | 1.072 |
|  | Constant | -1.645 | .016 | 11077.235 | 1 | .000 | .193 |  |  |
| a. Sex = Girl | | | | | | | | | |
| b. Variable(s) entered on step 1: year 2021, family’s financial situation, year 2021 * family’s financial situation. | | | | | | | | | |

Table S11. Logistic regression model with COVID-19 (year 2021; year 2019 as the reference category), family’s financial situation (”Good” as the reference category), and their interaction predicting generalized anxiety (GAD-7 ≥ 10) in boys.

| **Variables in the Equation^a^** | | | | | | | | | |
| --- | --- | --- | --- | --- | --- | --- | --- | --- | --- |
|  | | B | S.E. | Wald | df | Sig. | Exp(B) | 95% C.I.for EXP(B) | |
|  |  |  |  |  |  |  |  | Lower | Upper |
| Step 1^b^ | Year 2021 | .485 | .040 | 150.586 | 1 | .000 | 1.625 | 1.503 | 1.756 |
|  | Family's financial situation |  |  | 601.748 | 2 | .000 |  |  |  |
|  | Family's financial situation (Moderate) | .818 | .055 | 220.918 | 1 | .000 | 2.266 | 2.034 | 2.524 |
|  | Family's financial situation (Poor) | 1.714 | .076 | 502.350 | 1 | .000 | 5.550 | 4.777 | 6.447 |
|  | Year 2021 * Family's financial situation |  |  | 1.676 | 2 | .433 |  |  |  |
|  | Year 2021 by Family's financial situation (Moderate) | -.077 | .071 | 1.175 | 1 | .278 | .926 | .806 | 1.064 |
|  | Year 2021 by Family's financial situation (Poor) | -.094 | .101 | .860 | 1 | .354 | .910 | .746 | 1.110 |
|  | Constant | -3.324 | .031 | 11400.968 | 1 | .000 | .036 |  |  |
| a. Sex = Boy | | | | | | | | | |
| b. Variable(s) entered on step 1: year 2021, family’s financial situation, year 2021 * family’s financial situation. | | | | | | | | | |

Table S12. Logistic regression model with COVID-19 (year 2021; year 2019 as the reference category), family’s financial situation (”Good” as the reference category), and their interaction predicting generalized anxiety (GAD-7 ≥ 10) in girls.

| **Variables in the Equation^a^** | | | | | | | | | |
| --- | --- | --- | --- | --- | --- | --- | --- | --- | --- |
|  | | B | S.E. | Wald | df | Sig. | Exp(B) | 95% C.I.for EXP(B) | |
|  |  |  |  |  |  |  |  | Lower | Upper |
| Step 1^b^ | Year 2021 | .629 | .021 | 938.626 | 1 | .000 | 1.876 | 1.802 | 1.953 |
|  | Family's financial situation |  |  | 1418.725 | 2 | .000 |  |  |  |
|  | Family's financial situation (Moderate) | .762 | .028 | 756.207 | 1 | .000 | 2.142 | 2.029 | 2.262 |
|  | Family's financial situation (Poor) | 1.361 | .044 | 956.621 | 1 | .000 | 3.902 | 3.579 | 4.254 |
|  | Year 2021 * Family's financial situation |  |  | 9.911 | 2 | .007 |  |  |  |
|  | Year 2021 by Family's financial situation (Moderate) | -.107 | .036 | 8.698 | 1 | .003 | .898 | .837 | .965 |
|  | Year 2021 by Family's financial situation (Poor) | -.101 | .062 | 2.684 | 1 | .101 | .904 | .801 | 1.020 |
|  | Constant | -1.724 | .016 | 11638.783 | 1 | .000 | .178 |  |  |
| a. Sex = Girl | | | | | | | | | |
| b. Variable(s) entered on step 1: year 2021, family’s financial situation, year 2021 * family’s financial situation. | | | | | | | | | |

Table S13. Logistic regression model with COVID-19 (year 2021; year 2019 as the reference category), sum-index of socioeconomic adversities (0 as the reference category), and their interaction predicting depression (PHQ-2 ≥ 3) in boys.

| **Variables in the Equation^a^** | | | | | | | | | |
| --- | --- | --- | --- | --- | --- | --- | --- | --- | --- |
|  | | B | S.E. | Wald | df | Sig. | Exp(B) | 95% C.I.for EXP(B) | |
|  |  |  |  |  |  |  |  | Lower | Upper |
| Step 1^b^ | Year 2021 | .369 | .040 | 84.851 | 1 | .000 | 1.446 | 1.337 | 1.564 |
|  | Sum-index of socioeconomic  adversities |  |  | 753.316 | 3 | .000 |  |  |  |
|  | Sum-index of socioeconomic  adversities (1) | .582 | .045 | 164.671 | 1 | .000 | 1.790 | 1.638 | 1.956 |
|  | Sum-index of socioeconomic  adversities (2) | 1.133 | .058 | 383.712 | 1 | .000 | 3.105 | 2.772 | 3.478 |
|  | Sum-index of socioeconomic  adversities (3-4) | 1.931 | .086 | 502.403 | 1 | .000 | 6.898 | 5.826 | 8.167 |
|  | Year 2021 * Sum-index of socioeconomic  adversities |  |  | 4.394 | 3 | .222 |  |  |  |
|  | Year 2021 by Sum-index of socioeconomic  adversities (1) | -.096 | .060 | 2.567 | 1 | .109 | .909 | .809 | 1.022 |
|  | Year 2021 by Sum-index of socioeconomic  adversities (2) | -.012 | .076 | .027 | 1 | .869 | .988 | .851 | 1.146 |
|  | Year 2021 by Sum-index of socioeconomic  adversities (3-4) | -.183 | .117 | 2.440 | 1 | .118 | .833 | .662 | 1.048 |
|  | Constant | -2.882 | .031 | 8788.037 | 1 | .000 | .056 |  |  |
| a. Sex = Boy | | | | | | | | | |
| b. Variable(s) entered on step 1: year 2021, sum-index of socioeconomic adversities, year 2021 * sum-index of socioeconomic adversities. | | | | | | | | | |

Table S14. Logistic regression model with COVID-19 (year 2021; year 2019 as the reference category), sum-index of socioeconomic adversities (0 as the reference category), and their interaction predicting depression (PHQ-2 ≥ 3) in girls.

| **Variables in the Equation^a^** | | | | | | | | | |
| --- | --- | --- | --- | --- | --- | --- | --- | --- | --- |
|  | | B | S.E. | Wald | df | Sig. | Exp(B) | 95% C.I.for EXP(B) | |
|  |  |  |  |  |  |  |  | Lower | Upper |
| Step 1^b^ | Year 2021 | .533 | .025 | 455.345 | 1 | .000 | 1.704 | 1.623 | 1.790 |
|  | Sum-index of socioeconomic  adversities |  |  | 1394.725 | 3 | .000 |  |  |  |
|  | Sum-index of socioeconomic  adversities (1) | .490 | .029 | 292.478 | 1 | .000 | 1.632 | 1.543 | 1.727 |
|  | Sum-index of socioeconomic  adversities (2) | 1.087 | .035 | 947.665 | 1 | .000 | 2.965 | 2.766 | 3.177 |
|  | Sum-index of socioeconomic  adversities (3-4) | 1.514 | .058 | 683.800 | 1 | .000 | 4.546 | 4.058 | 5.092 |
|  | Year 2021 * Sum-index of socioeconomic  adversities |  |  | 1.939 | 3 | .585 |  |  |  |
|  | Year 2021 by Sum-index of socioeconomic  adversities (1) | -.021 | .037 | .313 | 1 | .576 | .979 | .910 | 1.054 |
|  | Year 2021 by Sum-index of socioeconomic  adversities (2) | -.065 | .047 | 1.916 | 1 | .166 | .937 | .854 | 1.028 |
|  | Year 2021 by Sum-index of socioeconomic  adversities (3-4) | -.009 | .081 | .012 | 1 | .914 | .991 | .845 | 1.162 |
|  | Constant | -1.719 | .019 | 8035.520 | 1 | .000 | .179 |  |  |
| a. Sex = Girl | | | | | | | | | |
| b. Variable(s) entered on step 1: year 2021, sum-index of socioeconomic adversities, year 2021 * sum-index of socioeconomic adversities. | | | | | | | | | |

Table S15. Logistic regression model with COVID-19 (year 2021; year 2019 as the reference category), sum-index of socioeconomic adversities (0 as the reference category), and their interaction predicting generalized anxiety (GAD-7 ≥ 10) in boys.

| **Variables in the Equation^a^** | | | | | | | | | |
| --- | --- | --- | --- | --- | --- | --- | --- | --- | --- |
|  | | B | S.E. | Wald | df | Sig. | Exp(B) | 95% C.I.for EXP(B) | |
|  |  |  |  |  |  |  |  | Lower | Upper |
| Step 1^b^ | Year 2021 | .470 | .050 | 88.598 | 1 | .000 | 1.601 | 1.451 | 1.765 |
|  | Sum-index of socioeconomic  adversities |  |  | 490.051 | 3 | .000 |  |  |  |
|  | Sum-index of socioeconomic  adversities (1) | .488 | .059 | 69.050 | 1 | .000 | 1.629 | 1.452 | 1.828 |
|  | Sum-index of socioeconomic  adversities (2) | 1.157 | .071 | 263.678 | 1 | .000 | 3.181 | 2.766 | 3.657 |
|  | Sum-index of socioeconomic  adversities (3-4) | 1.862 | .103 | 328.294 | 1 | .000 | 6.440 | 5.265 | 7.877 |
|  | Year 2021 * Sum-index of socioeconomic  adversities |  |  | .351 | 3 | .950 |  |  |  |
|  | Year 2021 by Sum-index of socioeconomic  adversities (1) | -.022 | .075 | .090 | 1 | .765 | .978 | .844 | 1.132 |
|  | Year 2021 by Sum-index of socioeconomic  adversities (2) | -.053 | .092 | .334 | 1 | .563 | .948 | .792 | 1.135 |
|  | Year 2021 by Sum-index of socioeconomic  adversities (3-4) | -.007 | .134 | .003 | 1 | .958 | .993 | .764 | 1.291 |
|  | Constant | -3.426 | .039 | 7616.955 | 1 | .000 | .033 |  |  |
| a. Sex = Boy | | | | | | | | | |
| b. Variable(s) entered on step 1: year 2021, sum-index of socioeconomic adversities, year 2021 * sum-index of socioeconomic adversities. | | | | | | | | | |

Table S16. Logistic regression model with COVID-19 (year 2021; year 2019 as the reference category), sum-index of socioeconomic adversities (0 as the reference category), and their interaction predicting generalized anxiety (GAD-7 ≥ 10) in girls.

| **Variables in the Equation^a^** | | | | | | | | | |
| --- | --- | --- | --- | --- | --- | --- | --- | --- | --- |
|  | | B | S.E. | Wald | df | Sig. | Exp(B) | 95% C.I.for EXP(B) | |
|  |  |  |  |  |  |  |  | Lower | Upper |
| Step 1^b^ | Year 2021 | .641 | .025 | 650.721 | 1 | .000 | 1.899 | 1.808 | 1.995 |
|  | Sum-index of socioeconomic  adversities |  |  | 1192.503 | 3 | .000 |  |  |  |
|  | Sum-index of socioeconomic  adversities (1) | .475 | .029 | 263.488 | 1 | .000 | 1.608 | 1.519 | 1.703 |
|  | Sum-index of socioeconomic  adversities (2) | 1.013 | .036 | 789.196 | 1 | .000 | 2.754 | 2.566 | 2.956 |
|  | Sum-index of socioeconomic  adversities (3-4) | 1.436 | .058 | 607.252 | 1 | .000 | 4.205 | 3.751 | 4.714 |
|  | Year 2021 * Sum-index of socioeconomic  adversities |  |  | 10.564 | 3 | .014 |  |  |  |
|  | Year 2021 by Sum-index of socioeconomic  adversities (1) | -.095 | .038 | 6.251 | 1 | .012 | .910 | .845 | .980 |
|  | Year 2021 by Sum-index of socioeconomic  adversities (2) | -.135 | .048 | 7.950 | 1 | .005 | .874 | .796 | .960 |
|  | Year 2021 by Sum-index of socioeconomic  adversities (3-4) | -.053 | .081 | .426 | 1 | .514 | .949 | .809 | 1.112 |
|  | Constant | -1.793 | .020 | 8379.957 | 1 | .000 | .166 |  |  |
| a. Sex = Girl | | | | | | | | | |
| b. Variable(s) entered on step 1: year 2021, sum-index of socioeconomic adversities, year 2021 * sum-index of socioeconomic adversities. | | | | | | | | | |
